# Deep Eutectic Solvents for Subcutaneous Delivery of Protein Therapeutics

**DOI:** 10.1002/advs.202205389

**Published:** 2023-01-15

**Authors:** Alexander M. Curreri, Jayoung Kim, Michael Dunne, Pavimol Angsantikul, Morgan Goetz, Yongsheng Gao, Samir Mitragotri

**Affiliations:** ^1^ John A. Paulson School of Engineering and Applied Sciences Harvard University 150 Western Ave Allston MA 02134 USA; ^2^ Wyss Institute for Biologically Inspired Engineering at Harvard University 3 Blackfan St Boston MA 02115 USA; ^3^ Present address: The Population Council One Dag Hammarskjold Plaza New York NY 10017 USA

**Keywords:** bioavailability, biologics, deep eutectic solvents, ionic liquids, monoclonal antibodies, pharmacokinetics, subcutaneous

## Abstract

Proteins are among the most common therapeutics for the treatment of diabetes, autoimmune diseases, cancer, and metabolic diseases, among others. Despite their common use, current protein therapies, most of which are injectables, have several limitations. Large proteins such as monoclonal antibodies (mAbs) suffer from poor absorption after subcutaneous injections, thus forcing their administration by intravenous injections. Even small proteins such as insulin suffer from slow pharmacokinetics which poses limitations in effective management of diabetes. Here, a deep eutectic‐based delivery strategy is used to offer a generalized approach for improving protein absorption after subcutaneous injections. The lead formulation enhances absorption of mAbs after subcutaneous injections by ≈200%. The same composition also improves systemic absorption of subcutaneously injected insulin faster than Humalog, the current gold‐standard of rapid acting insulin. Mechanistic studies reveal that the beneficial effect of deep eutectics on subcutaneous absorption is mediated by their ability to reduce the interactions of proteins with the subcutaneous matrix, especially collagen. Studies also confirm that these deep eutectics are safe for subcutaneous injections. Deep eutectic‐based formulations described here open new possibilities for subcutaneous injections of therapeutic proteins.

## Graphical abstract

Here the use of deep eutectic solvents in biologic formulations is reported to improve the pharmacokinetic properties of insulin and monoclonal antibodies. The underlying mechanisms are reported and the safety of deep eutectic biologics formulations in a rodent model is evaluated.

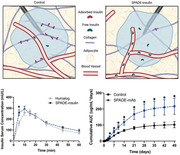

## 1 Introduction

Recombinant protein biologics are among the most extensively used therapeutics in the clinic over the past 40 years. In the past 8 years, protein biologics have accounted for around 30% of Food and Drug Administration (FDA) approvals.^[^ ^1^, ^2^ ^]^ Monoclonal antibodies (mAbs) and antibody conjugates, which recently saw their 100th approval and average around 10 approvals per year, make up a majority of the new protein biologic approvals.^[^ ^3^ ^]^ Delivery logistics of biologics, on the other hand, have seen limited innovation. Although many mAbs are delivered by intravenous (IV) administration, subcutaneous administration (SC) offers a better alternative to IV delivery by lowering treatment costs owing to reduced strain on healthcare resources,^[^ ^4^, ^5^ ^]^ decreasing injection pain and potential for infection,^[^ ^6^ ^]^ and improved patient compliance by providing self‐administration options.^[^ ^7^, ^8^, ^9^ ^]^ Despite these advantages, the use of subcutaneous injections for some mAbs is limited by their poor bioavailability.^[^ ^10^ ^]^ Subcutaneously administered biologics must traverse the subcutaneous tissue, comprising cellular milieu and extracellular matrix (ECM) proteins, before reaching the systemic circulation by absorption into the local blood or lymph capillaries.^[^ ^11^ ^]^ Smaller biologics, e.g., insulin monomers (MW = 6 kDa) drain into blood capillaries, whereas larger proteins such as insulin hexamers (MW = 36 kDa) and mAbs (MW = 150 kDa) drain into lymph capillaries.^[^ ^12^ ^]^ Significant mAb drug product is lost prior to lymph absorption via protease degradation and endocytosis in the subcutaneous space and in the lymph capillaries prior to thoracic duct trafficking where mAb molecules can enter the systemic circulation.^[^ ^13^ ^]^

Insulin and mAbs have been at the center of attention for development of methodologies to improve absorption after their subcutaneous injection, in particular via protein modification and formulation engineering. Substantial efforts have been focused on developing new insulin analogs to control the duration of action over a long, intermediate, short, or rapid time scales. Many academic research efforts have been focused on developing strategies for sustained insulin release, however, rapid‐acting insulin formulations remain relatively underexplored.^[^ ^14^ ^]^ Rapid‐acting insulin analogs are often used in conjunction with continuous infusion pumps to mitigate hyperglycemic episodes by decreasing the time between blood glucose measurements and insulin's systemic effect.^[^ ^15^ ^]^ A clinically approved insulin analog, Humalog (insulin lispro) exploits an amino acid sequence mutation to dissociate the stable insulin hexamers into insulin monomers in the subcutaneous space upon injection and formulation dilution, thus allowing for rapid absorption into the bloodstream.^[^ ^16^, ^17^, ^18^ ^]^ Protein engineering‐based approaches have also been attempted for improving mAb pharmacokinetics. For example, modifications of the Fc region have been attempted to increase mAb's subcutaneous absorption.^[^ ^19^, ^20^ ^]^

Formulation engineering, on the other hand, offers an alternate approach to control protein pharmacokinetics without protein design constraint. Efforts, apart from extended release systems, have been based on two principles: reduced protein aggregation and enzymatic degradation of the matrix in the subcutaneous compartment. Mann et al.^[^ ^21^ ^]^ developed a polymer excipient that reduced insulin aggregation and decreased the time to insulin peak in vivo after subcutaneous injections. These studies reported 64% faster insulin absorption compared to Humalog. Poly(ethylene glycol) (PEG)^[^ ^22^, ^23^, ^24^ ^]^ and trehalose glycopolymer^[^ ^25^ ^]^ conjugation have also been used to stabilize insulin monomers for rapid‐acting formulation but can have negative effects on pharmacokinetics. Efforts to improve systemic absorption of mAbs after subcutaneous injection have focused less on improving bioavailability and more on pushing formulation concentration and injection volume limits.^[^ ^26^ ^]^ One of the few exceptions is the use of recombinant hyaluronidase, an ECM‐degrading enzyme, coformulated with mAbs to improve the bioavailability.^[^ ^27^ ^]^ There have been 5 such FDA approvals: Rituxan HYCELA, Herceptin HYLECTA, DARZALEX Faspro, PHESGO.

Here we report the use of biocompatible deep eutectics to improve subcutaneous pharmacokinetics via a novel mechanism of action which adds a new tool toward improving subcutaneous formulations for biologics. Unlike many other biological barriers, transport barriers in the subcutaneous space are poorly understood. The subcutaneous ECM, which is most abundantly comprised of type I collagen, has the potential to limit systemic absorption of subcutaneously administered proteins through a variety of nonspecific binding interactions with the injected biologics.^[^ ^28^ ^]^ Since degradation in the subcutaneous space can play an important role in material loss,^[^ ^13^ ^]^ nonspecific binding between biologics and ECM proteins like type I collagen could increase subcutaneous residence time and make the therapeutics more susceptible to proteosome degradation and endocytosis. We hypothesized that subcutaneously injected protein formulations interact with the ECM proteins and such interactions lead to delayed or reduced absorption into systemic circulation. Reducing protein‐matrix interactions in the subcutaneous space can thus potentially increase systemic bioavailability. Here we report the ability of Ionic Liquids (ILs) and deep eutectic solvents (DESs) to accomplish this goal.

ILs and DESs are salt compounds comprised primarily of organic ions that exist in a liquid state below 100 °C. While ILs generally consist of cation: anion ratios of 1:1, DESs can possess varying ion ratios and have an additional requirement that their melting point is lower than those of their precursors. The unique ability and tunability of ILs to mediate interactions with biological milieu have opened a large number of biomedical applications including drug delivery, protein stabilization, and biosensing, among others.^[^ ^29^ ^]^ Taking advantage of the broad ability of ILs to mediate interactions in biological systems, we hypothesized that ionic liquids and deep eutectics can also mitigate the interactions of injected proteins with the proteins in the subcutaneous ECM. We refer to such approach as Subcutaneous Protein Administration using Deep Eutectics (SPADE). Here we report screening of SPADE formulations and their ability to improve subcutaneous injection speed and bioavailability for insulin and Rituximab, respectively. Our studies also demonstrate the lead SPADE formulation reduces protein interactions with collagen and is safe to inject as evidenced by repeat dose administration.

## 2 Results

### 2.1 Insulin Stabilization Using DES

Ten DESs were synthesized using a salt metathesis reaction at the cation:anion ratios of 1:2 as previously described.^[^ ^30^ ^]^ These DESs were designed to exhibit a range of chemical properties, especially hydrophobicity as this parameter is expected to be a key determinant of their ability to impact transport and subsequent absorption in the subcutaneous tissue. Choline and acetylcholine were investigated as the two cations because choline has been a commonly explored cation in previous studies of ionic liquids^[^ ^30^, ^31^, ^32^, ^33^, ^34^, ^35^, ^36^, ^37^ ^]^ and acetylcholine is structurally similar but a more hydrophobic cation. These cations were subsequently paired with five anions, glycolate, lactate, propionate, hexenoate, and geranate, that covered a spectrum of molecular weights, carbon chain lengths, and hydrophobicities (Table S1, Supporting Information). A library of ten DESs was synthesized: choline glycolate (CG), acetylcholine glycolate (aCG), choline lactate (CL), acetylcholine lactate (aCL), choline propionate (CP), acetylcholine propionate (aCP), choline hexenoate (CH), acetylcholine hexenoate (aCH), choline geranate (CAGE), and acetylcholine geranate (aCAGE). 1D proton nuclear magnetic resonance (NMR) was used to confirm their structures (see the Supporting Information). The ten DESs were then formulated as a 0.5% solution in sterile saline, which readily solubilized 100 U mL^−1^ regular insulin (commonly used concentration in clinical formulations) at neutral pH.

**Figure 1:**
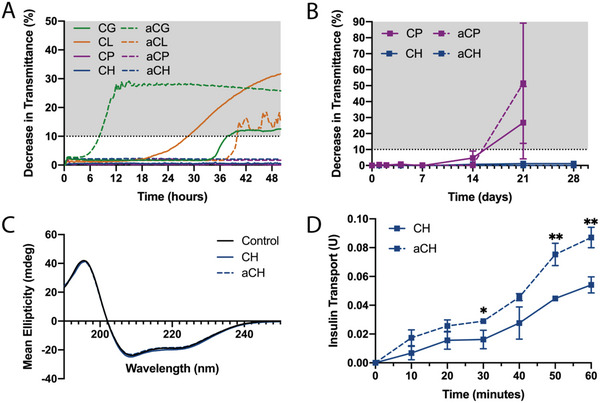
Determination of SPADE formulation. A) Average decrease in transmittance of insulin‐DES formulations stored at 37 °C with continuous shaking for 50 h. The shaded gray represents the region in which formulations were considered unstable (*n* = 3). B) Average decrease in transmittance of insulin‐DES formulations that were stable in (A) stored at 4 °C for 28 days. The shaded gray represents the region in which formulations were considered unstable (*n* = 3). C) Circular dichroism for insulin‐DES formulations that were stable in (B) compared to the fresh, stable insulin control (*n* = 5). D) Insulin transport across HUVEC monolayer on transwell cell culture inserts (*n* = 3). Data plotted as mean ± standard deviation (SD) and statistical significance was determined with a *t*‐test. *p < 0.05, **p < 0.01.

Successful dissolution of insulin was determined by the turbidity of the formulation, assessed by converting absorbance at 540 nm to transmittance.^[^ ^21^ ^]^ The percent transmittance threshold for the successful formulation was set at 80% as this was the mean transmittance for the clinical comparator (Humalog). All but two formulations met this criterion (Figure S1, Supporting Information). Low transmittance values are indicative of insoluble insulin aggregates that cannot be present in clinical formulations. The current guidance is that injectable formulations of 100 mL or less should have fewer than 6000 particles greater than or equal to 10 µm and less than 0.65% of protein molecules should be greater than 50 µm.^[^ ^38^, ^39^ ^]^ To further screen the remaining DES formulations, a stressed aging test was performed to determine their stability for 50 hours at 37 °C with constant shaking. Formulations that experienced a decrease in transmittance of greater than 10% at any point during the 50 hours period compared to their respective initial transmittance were considered unstable and were eliminated from contention. This transmittance decrease was seen for CG, aCG, CL, and aCL (**Figure** **1**A), leaving only four viable formulations (CP, aCP, CH, and aCH).

Additional stability screening was performed to assess cold‐chain stability and confirm monomer conformational stability. Cold‐chain, namely refrigeration between 2 and 8 °C, is critical for protein biologic stability to prevent unfolding, aggregation, and is widely used to extend shelf‐life.^[^ ^40^ ^]^ Among CP, aCP, CH, and aCH formulations incubated at 4 °C over 28 days, both propionate‐based DES formulations experienced aggregation based on a greater than 10% decrease in transmittance between Day 14 and 21, while both hexenoate‐based DESs were stable through Day 28 (Figure 1B). The stability of hexenoate‐based formulations was further confirmed by assessing the protein's secondary structure through circular dichroism (CD). The CD spectra of insulin incubated with the two leading formulations, CH and aCH, at 37 °C for 2 hours matched that of the control insulin dissolved in sodium phosphate buffer, suggesting that neither DES formulation induced notable protein unfolding at physiological temperature and pharmacologically relevant time scales (Figure 1C). CD spectra integrity was also confirmed by measuring the high‐tension voltage, which was maintained below 500 V for the relevant insulin CD wavelengths (Figure S2, Supporting Information). Insulin in both the CH and aCH formulations were confirmed to have stable secondary structures following the same stressed aging and cold chain storage conditions (7 and 21 days) (Figure S3, Supporting Information).

To further select the formulation from two leads, CH and aCH, their effect on trans‐endothelial transport of insulin was measured in vitro. Endothelial cells of blood capillaries provide a barrier to vascular drainage and a beneficial effect of DESs on trans‐endothelial transport can further improve pharmacokinetics. To assess this possibility, in vitro transport of insulin across human umbilical vein endothelial cell (HUVEC) was measured using transwell permeability assay. 0.15% v/v or ≈4.3 mm DES was used in these studies based on the in vitro tolerability study (Figure S4, Supporting Information). HUVEC monolayers were formed on gelatin‐coated transwell and confirmed by fluorescent imaging with Hoescht 33 342 (nuclei) and Actin 488 (cell cytoskeleton) (Figure S5, Supporting Information). aCH exhibited enhanced vascular permeability compared to CH (Figure 1D). The combination of stability and transport experiments led to the choice of aCH as the lead composition for subsequent studies.

### 2.2 SPADE Prevents Interactions Between Therapeutic Proteins and ECM Collagen

**Figure 2:**
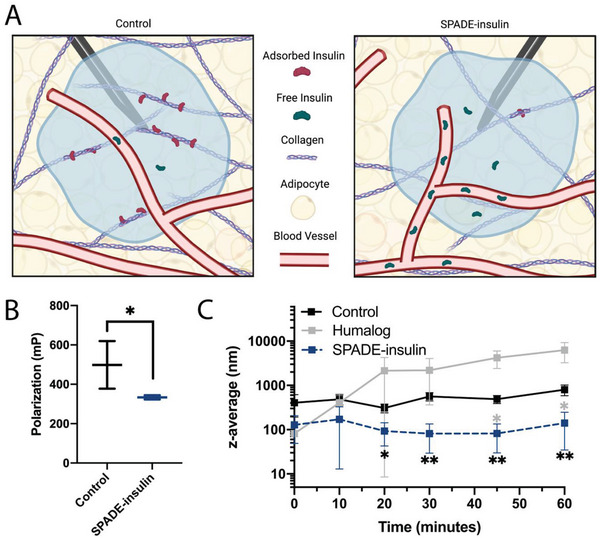
SPADE mechanism of action. A) A schematic representation of SPADE‐insulin (right) as compared to the non‐DES‐containing control (left) when administer in the subcutaneous space. B) Fluorescence polarization for the control (insulin formulated in saline) and SPADE‐insulin when mixed with collagen (Control *n* = 6, SPADE‐insulin *n* = 5). C) Average diameter as measured with DLS versus time of control (insulin formulated in saline), Humalog, and SPADE‐insulin when mixed with collagen (*n* = 3). Data plotted as mean ± standard deviation (SD) and statistical significance was determined with a *t*‐test. *p < 0.05, **p <0.01. Asterisks below the SPADE‐insulin (blue) points refer to statistical comparison to the control (black) and above the SPADE‐insulin points refer to statistical comparison to Humalog (gray).

The ability of SPADE to mitigate the interactions of proteins with ECM was assessed using the lead DES, aCH. A schematic representation of the hypothesized mechanism is depicted in **Figure** **2**A. SPADE (right) is hypothesized to decrease nonspecific binding between administered therapeutic proteins and subcutaneous extracellular proteins like collagen to allow for faster and greater absorption of protein biologics. To test this hypothesis, we performed fluorescence polarization (FP) and utilized dynamic light scattering (DLS) to determine the degree of collagen‐insulin association. FP is commonly used as a ligand‐binding assay to analyze interactions between small molecules or peptide payloads binding to their protein targets,^[^ ^41^, ^42^ ^]^ based on the principle that a protein‐bound, fluorescently‐labeled molecule rotates more slowly and emits in the parallel direction, resulting in a higher polarization value.^[^ ^43^ ^]^ FP was performed immediately after mixing Cy5.5‐labeled insulin, either formulated in aCH or saline (control), and Type I human collagen. The resulting polarization values showed that aCH inhibits nonspecific insulin‐collagen binding compared to the control (Figure 2B). The average polarization was 1.5 times higher for the control group than the SPADE group. To further confirm the FP results and compare against Humalog, DLS was used to measure the average particle diameter (z‐avg) after mixing insulin formulations with collagen, as the z‐avg for insulin‐bound collagen would increase with fewer unbound insulin molecules remaining and only the collagen or collagen‐insulin complex being detected. The initial average z‐avg for both the Humalog‐ and SPADE‐insulin‐collagen mixtures were ≈100 nm, while the control‐collagen was around 400 nm. Consistent with the FP results, the initial larger average size of the control insulin‐collagen mixture suggests that the insulin in this formulation binds almost immediately to collagen in solution. The Humalog group exhibited an increase in z‐avg for the entire time course, reaching a value exceeding 5000 nm over 60 minutes. In contrast, SPADE‐insulin group remained at approximately 100 nm over the same time period (Figure 2C). The results from FP and DLS strongly support that the SPADE reduces nonspecific binding between insulin molecules and collagen that is ubiquitously present in the subcutaneous space as part of the ECM.

### 2.3 SPADE Enhances Insulin Pharmacokinetics

**Figure 3:**
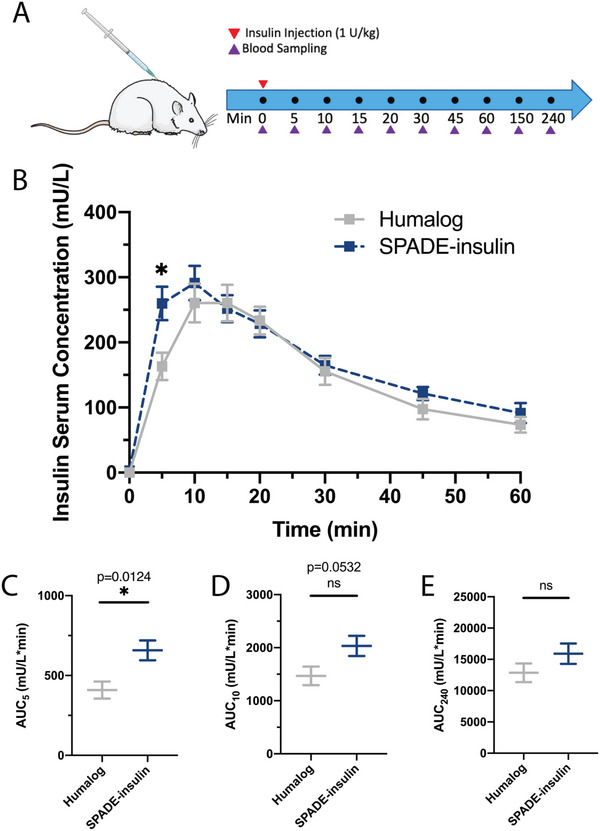
SPADE‐insulin pharmacokinetics. A) Pharmacokinetic study design for SPADE‐insulin versus Humalog including subcutaneous injections of 1 U kg^−1^ insulin (red arrow) and blood sampling schedule (purple arrow). B) Insulin serum concentration against time for the first 60 minutes of the pharmacokinetic study. C) AUC 5 minutes after injection. D) AUC 10 minutes after injection. E) AUC 240 minutes after injection. (Humalog *n* = 6, SPADE‐insulin *n* = 5) Statistical significance was determined with a *t*‐test. *p < 0.05. The Figure was partly generated using Servier Medical Art, provided by Servier, licensed under a Creative Commons Attribution 3.0 unported license.

Pharmacokinetics of 1 U kg^−1^ SPADE‐insulin was compared against the equivalent dose of Humalog, the clinical gold standard of fast‐acting insulin analog, in rats. The study design is detailed in **Figure** **3**A. SPADE‐insulin significantly accelerated insulin absorption from the subcutaneous tissue compared to Humalog (Figure 3B), as shown by 1.6‐fold higher serum level of insulin at 5 minutes after injection of SPADE‐insulin compared to Humalog. In addition, the area under the curve (AUC) was calculated at each time point to quantify the accumulation of insulin in the bloodstream. The cumulative AUC also increased significantly at 5 minutes by 1.6‐fold in SPADE‐insulin ‐treated rats compared to Humalog‐injected rats (Figure 3C). At 10 minutes, the difference between the cumulative AUC in the SPADE‐insulin and Humalog was negligible (Figure 3D) and none at other times (Figure 3D; and Figure S6, Supporting Information). Statistically significantly higher absorption of insulin at an early time point of 5 minutes suggests that aCH in SPADE‐insulin plays an important role immediately after the administration and could benefit patients in need of ultrafast insulin that ensures less time delay to therapeutic effect. Additionally, while these studies show stability of SPADE‐insulin in rat serum, stability in human serum was also confirmed (Figure S7, Supporting Information).

### 2.4 SPADE is Nontoxic and Safe Upon Repeat Dosing

**Figure 4:**
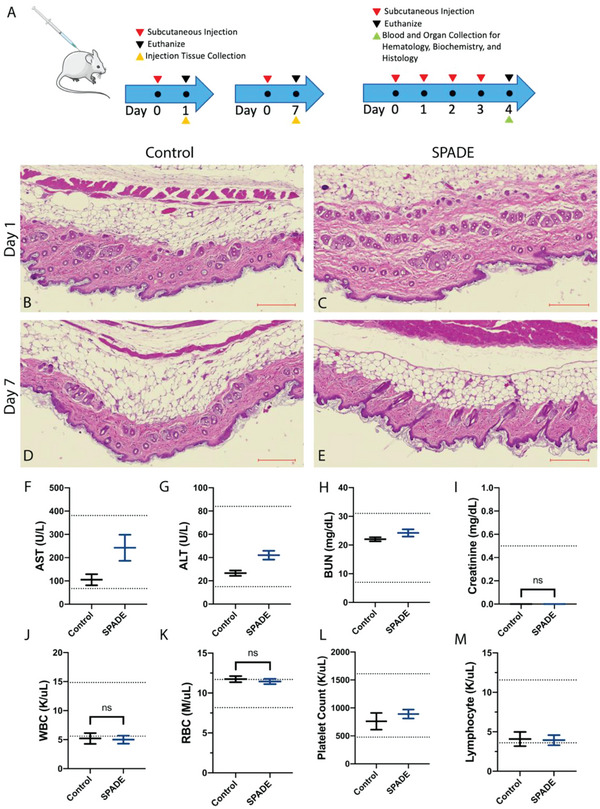
SPADE safety assessment. A) Safety study designs for SPADE versus control (saline) including injection site toxicity (left) and repeat dosing study (right). The studies incorporated subcutaneous injection (red arrow), injection site tissue collection (yellow arrow), blood and vital organ collection (green arrow), and euthanization (black arrow). B–E) Injection site H&E images for indicated formulation and time points. Scale bars, 200 µm. F) aspartate aminotransferase (AST) serum concentrations. G) Alanine aminotransferase (ALT) serum concentrations groups. H) Blood urea nitrogen (BUN) serum concentrations. I) Creatinine serum concentrations. J) White blood cell (WBC) counts. K) Red blood cell (RBC) counts. L) Platelet counts. M) Lymphocyte counts for control (*n* = 4) and SPADE (*n* = 5) treated groups. Dotted lines represent the expected range for metric of interest.^[^ ^45^ ^]^ Statistical significance was determined with a *t*‐test. *p < 0.05. The Figure was partly generated using Servier Medical Art, provided by Servier, licensed under a Creative Commons Attribution 3.0 unported license.

Two different studies were performed using saline (control) and SPADE‐alone dosed BALB/c mice to examine local (injection site) and systemic toxicities. The first study used four cohorts to examine injection site toxicity 24 hours and 7 days following subcutaneous dosing using hematoxylin and eosin staining (H&E) (**Figure** **4**A). There was no notable difference between the injection sites of the two formulations for either time point (Figure 4B–E). There is no noticeable difference between hematoxylin staining or nuclei pattern when comparing SPADE and control injection site sections.^[^ ^44^ ^]^ Additionally, a blind analysis of the tissue samples by a histopathologist confirmed no signs of toxicity, with respect to inflammation, edema, necrosis, fibrosis, or degeneration.

We then examined systemic toxicity from repeat dosing with SPADE. Blood and major organs were analyzed 24 hours after the last of four daily subcutaneous injections of saline (control) and SPADE alone formulations. Two key liver function markers, aspartate transaminase (AST) and alanine transaminase (ALT), were within the accepted range (Figure 4F–G).^[^ ^45^ ^]^ AST and ALT are both transfer enzymes found in hepatocyte cytoplasm that are released into the serum upon hepatocyte damage, thus levels elevated outside the expected range indicate improper liver function.^[^ ^46^ ^]^ Additionally, two key kidney function markers, blood urea nitrogen (BUN), and creatinine, were also within the accepted range for female BALB/c mice (Figure 4H,I).^[^ ^45^ ^]^ Urea and creatinine are both filtered out of serum by the glomeruli of the kidneys, thus BUN and creatinine serum levels are markers for glomerular filtration rate and elevated levels indicate kidney dysfunction.^[^ ^47^ ^]^ Other biochemical markers for SPADE‐treated mice were either within the accepted range or not statistically different from the saline control (Figure S8, Supporting Information). Whole blood analysis showed that SPADE is well‐tolerated and that no significant systemic immune response occurred upon repeat dosing. White blood cell, red blood cell, platelet, and lymphocyte counts were all within the accepted range or ones that were outside were not statistically different from the saline control (Figure 4J–M). Other peripheral cells measured in this study were within the accepted range or not statistically different from the saline control (Figure S9, Supporting Information). Systemic toxicity was also assessed by histopathology of mice who received repeat SPADE dosing. H&E staining showed no marked difference between SPADE and control mice in vital organs, including spleen, lung, kidney, liver, and heart (Figure S10, Supporting Information). These finds were also confirmed by a blind analysis by a histopathologist.

### 2.5 SPADE Enhances Bioavailability of Rituximab

**Figure 5:**
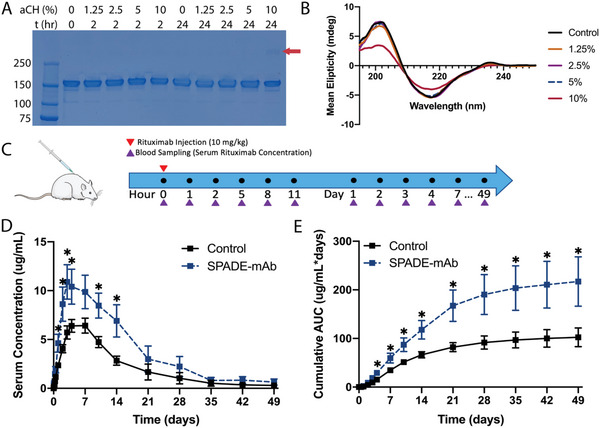
SPADE‐mAb stability, pharmacokinetics, and bioavailability. A) SDS‐PAGE gel electrophoresis for the assessment of SPADE‐mAb stability for indicated DES concentrations and 37 °C incubation times. The protein ladder is labeled with corresponding molecular weights (kDa). Red arrows indicate antibody aggregates. B) Circular dichroism spectra for 24 hours incubated formulations from (A) versus a control stable antibody formulation. C) Pharmacokinetic study design for SPADE‐mAb versus control including subcutaneous injections of 10 mg kg^−1^ rituximab (red arrow) and blood sampling schedule (purple arrow). D) Rituximab serum concentration versus time and E) AUC versus time for the 49 day antibody pharmacokinetic study for control (Rituximab biosimilar formulation, *n* = 6) and SPADE‐mAb (*n* = 5 until day 21, *n* = 4 day 28–49). Statistical significance was determined with a *t*‐test. *p < 0.05. The Figure was partly generated using Servier Medical Art, provided by Servier, licensed under a Creative Commons Attribution 3.0 unported license.

We explored whether SPADE can also enhance the absorption of a large protein biologic, namely the monoclonal antibody rituximab (SPADE‐mAb), that would benefit from greater absorption from subcutaneous administration. Due to the clinical requirement of higher dosing for subcutaneous antibody formulation compared to insulin, we first assessed DES concentration for stable SPADE formulation with rituximab using SDS‐PAGE and CD. Formulations were prepared with various DES concentrations and consistent rituximab concentrations and incubated at 37 °C for 2 and 24 h. After incubation, the samples were dialyzed against sodium phosphate to remove DES, and the resulting samples were diluted to the same concentration via UV–vis spectrophotometry before SDS‐PAGE and CD analyses. We determined that all DES formulation concentrations were stable apart from 10% v/v DES, as there is a faint band further up the gel that is indicative of higher‐order molecular weight species from potential aggregation (**Figure** **5**A). To further confirm this CD was used to assess the secondary structure of the samples that were incubated for 24 h. The CD results were consistent with the SDS‐PAGE results as the 10% v/v DES formulation saw a shift in mean ellipticity when compared to the control, while none of the other DES concentrations experienced this shift (Figure 5B). This shift is indicative of misfolding that likely caused the aggregation seen in the SDS‐PAGE gel. These stability studies indicated 5% v/v aCH as the appropriate concentration for the SPADE‐mAb formulation. To confirm that injectability would not be altered by DES, viscosities of the saline‐Ab and SPADE‐Ab formulations were measured using human IgG as a surrogate. The SPADE‐Ab viscosity was lower at every shear rate tested compared to saline‐Ab (Figure S11, Supporting Information).

Following the experimental design shown in Figure 5C, we performed a pharmacokinetics study and determined serum rituximab levels quantified by Enzyme‐linked immunosorbent assay (ELISA). Serum rituximab levels for SPADE were significantly higher 1 hours after administration and remained so through day 14, apart from 11 hours and 7 days (Figure 5D). Average peak rituximab serum concentration was higher, 10.89 compared to 6.41 µg mL^−1^, and occurred earlier, day 3 compared to day 7, for the SPADE‐mAb group than the control. AUC was also statistically higher at all time points of the study (Figure 5E). The significance values for each timepoint can be found in Table S2 (Supporting Information). At the study endpoint, AUC for SPADE treated group was 217.11 mg mL^−1^ *day, which is 2.12 times the control. AUC also had a higher fold increase over the control at early time points compared to later time points, suggesting that SPADE‐mAb is also improving the early absorption kinetics of antibodies as in the case of SPADE‐insulin (Figure S12, Supporting Information).

## 3 Discussion

Fast‐acting insulin reduces the time between blood glucose measurement and insulin's systemic effect, and it improves the ability to maintain euglycemia. This could make a significant difference especially for patients who use continuous subcutaneous insulin infusion (insulin pumps) that allows blood glucose level within the target ranges for a higher percentage of the day.^[^ ^15^ ^]^ While insulin analogs have been used to improve absorption kinetics in the clinic, significant research in academia has also focused on polymer^[^ ^21^ ^]^ and polypeptide^[^ ^48^ ^]^ excipients, polymer conjugation,^[^ ^24^ ^]^ and protein coformulation.^[^ ^49^ ^]^ While these strategies have shown success in vivo, DESs and ILs offer an appealing alternative since they can easily be manufactured at scale with fewer steps and lower costs to allow for simpler fast‐acting insulin formulation.

Beyond reducing protein–collagen interactions, SPADE formulations have the potential to decrease protein–protein interactions within the formulation at certain DES conditions. This is perhaps less critical for therapeutic proteins like insulin where the clinical formulations are relatively dilute (e.g., 3.47 mg mL^−1^ or 100 U mL^−1^). However, such interactions can play a critical role in mAb formulations which deploy a much higher concentration, e.g., greater than or equal to 100 mg mL^−1^. SPADE could provide a novel tool to reduce mAb–mAb interactions and improve stability of mAb formulations. Further studies should explore this possibility.

SPADE increased rituximab absorption (measured as AUC) by 112% over saline formulated rituximab. In a study performed by Kagan et al.,^[^ ^27^ ^]^ rats dosed dorsally with 10 mg kg^−1^ of rituximab, hyaluronidase was used to increase rituximab absorption by 91%. This high efficacy of SPADE supports its potential clinical use since similar hyaluronidase is already in clinical use for multiple mAbs. Further, SPADE demonstrated the ability to increase absorption over a wide range of protein biologic sizes, 5.8–150 kDa, and thus may offer a subcutaneous formulation strategy for other protein biologics, peptides, and nucleic acids.

## 4 Conclusion

Here, we developed a deep eutectic solvent‐based formulation strategy, SPADE, to improve subcutaneous delivery of therapeutic protein biologics. We demonstrated that SPADE: i) can be stably formulated with insulin and antibodies, ii) is a safe, non‐toxic formulation strategy, and iii) improves pharmacokinetics of insulin and the bioavailability of monoclonal antibodies by preventing nonspecific binding interactions between therapeutic proteins and collagen ECM proteins. SPADE utilized a deep eutectic solvent that can be synthesized in a facile manner, allowing for a simple and scalable formulation. SPADE is prepared from biocompatible ions with known history of human exposure, thus improving its safety profile.

## 5 Experimental Section

### Materials and Cell Lines

Choline bicarbonate (80% w/w in water), acetylcholine chloride (≥ 99%), lactic acid, glycolic acid (99%), propionic acid (≥ 99.5%), trans‐2‐hexenoic acid (≥ 98%), geranic acid (85%), regular lyophilized insulin (Lot: 22B284), gelatin powder, Millicell Transwell inserts, hydrochloric acid (1 N), IgG from human serum (I4506 Lot: SLCF7655), Human Serum (H4522 Lot: SLCF0688) and sodium hydroxide (≥ 98%) were all purchased from Sigma‐Aldrich (St. Louis, MO). 0.2 m sodium phosphate buffer was purchased from Boston BioProducts, Inc. (Milford, MA). HUVEC cells (Passage Number 2), growth media, and additives were purchased from ATCC (Manassas, VA). CellTiter 96 Aqueous One Solution Cell Proliferation Assay (MTS) was purchased from Promoega (Madison, WI). Type I Human Collagen was purchased from Advanced BioMatrix (Carlsbad, CA). Cy 5.5 fluorescently‐labeled insulin was purchased from Nanocs Inc. (New York, NY). SDS‐PAGE Ladder (Precision Plus Protein All Blue Prestained Protein Standards #1 610 373), lamelli buffer, Tris/Glycine/SDS Running Buffer, Coomassie blue stain, Mini‐PROTEAN Tetra Vertical Electrophoresis Cell, and power supply were all purchased from Bio‐Rad Life Sciences (Hercules, CA). Rituximab Biosimilar (SIM008 Lot: 803121F1) was purchased from BioXCell (Lebanon, NH). Insulin ELISA (10‐1113‐01 Lot: 32 226) and Insulin Lispro NL‐ELISA (10‐1291‐01 Lot: 31 214) were purchased from Mercodia Inc. (Winston Salem, NC). Rituximab ELISA (KBI1010 Lot: RTM0821‐2) was purchased from Eagle Biosciences (Amherst, NH).

### Deep Eutectic Solvent Synthesis and Formulation Preparation

Deep eutectic solvents were synthesized as previously described.^[^ ^30^, ^37^ ^]^ Briefly, weak acids of desired anions were dissolved in minimal ultrapure water in a round bottom flask. The solution was heated, with stirring, using an oil bath to 40 °C in the case of the choline‐based DESs and 65 °C in the case of the acetylcholine‐based DESs. To prevent round bottom flask over‐flow, the cationic precursor was slowly added at a ratio of 1:2 cation: anion. The mixture was allowed to fully react overnight. Excess water was removed first by rotary evaporator for 3 hours then by vacuum oven set to 60 °C for 2 days. The structure was then confirmed using nuclear magnetic resonance (Bruker AVANCE NEO 400).^[^ ^31^, ^37^ ^]^ To prepare insulin formulations, lyophilized insulin was suspended in saline. Sufficient DES was added and in cases where this addition did not dissolve insulin, 1 m hydrochloric acid was added to adjust the pH to 2.5–3 and the solution became clear. To adjust the pH to between 7.0 and 7.5, 1 m sodium hydroxide was added. The formulation was adjusted by adding saline to give final insulin and DES concentrations of 100 U mL^−1^ and 0.5% v/v), respectively. To prepare antibody formulations, ≈40% v/v solutions of DESs in 0.9% saline were pH adjusted to 7.0–7.5 using 10 m sodium hydroxide. The DES solution was then formulated with Rituximab biosimilar stock (9.3 mg mL^−1^) and/or saline to give final concentrations of 7.9 mg mL^−1^ antibody and the indicated concentration of DES. For viscosity experiments, formulations were prepared with IgG from human serum instead of Rituximab biosimilar.

### Stability Evaluation of Insulin‐DES Formulations Using Transmittance

Transmittance was used to assess insulin‐DES formulation stability. Absorbance measurements taken at a wavelength of 540 nm with a BioTek Synergy neo2 Plate reader were converted to percent transmittance with Equation (1)

(1) $$T\left(\%\right)\;=10^{\left(2-A_{540}\right)}$$

This equation relates absorbance to percent transmittance, derived from the Beer–Lambert Law.

The insulin‐DES formations were first analyzed for initial transmittance to confirm that there was no insulin aggregation immediately after formulation preparation. Subsequently, formulations that passed initial screening were plated in a black‐walled 96 well plate (replicates of three) and subjected to a stressed aging assay in which the plate was incubated at 37 °C with constant shaking. Absorbance readings were taken every 15 minutes for 50 hours to assess the change in transmittance over time. For cold chain stability, formulations were tested in a similar manner, without constant shaking, at 4 °C for 28 days.

### Protein Stability Assessment Using Circular Dichroism and SDS‐PAGE

Insulin formulations were incubated at 37 °C for 1 h, 37 °C for 48 hours with shaking, and 4 °C for 7 and 21 days. Antibody formulations were incubated at 37 °C for 2 and 24 h. These samples were subsequently dialyzed against 10 mm Sodium Phosphate pH 7.4. In preparation for circular dichroism (CD) or SDS‐PAGE, the postdialysis concentration was measured with UV Spectrophotometry (Thermo Scientific NanoDrop One) and adjusted to 200 µg mL^−1^. CD was performed by loading quartz cuvettes (Starna Cells Spectrosil Quartz 1‐Q‐1), with 200 µL of the sample, into a CD spectrophotometer (Jasco J‐815). Mean ellipticity was measured from 190 to 250 nm.

To assess antibody aggregation, SDS‐PAGE was performed using vertical gel electrophoresis (BioRad Mini‐PROTEAN Tetra Cell) loaded with precast polyacrylamide gels (BioRad TGX 4–15%) according to the manufacturer protocol. To form SDS complexes, samples were incubated in 1X Lammeli buffer for 10 minutes at 70 °C before loading in the gel. After 30 minutes of electrophoresis, the gel was removed, and protein bands stained with Coomassie dye.

### Transwell Vascular Permeability Studies

To calculate the maximum concentration at which there was no cell death a MTS cell viability assay was performed. HUVECs were cultured according to supplier protocols, subcultured, and seeded in a 96 well‐plate at a density of 10 000 cells per well. After allowing for cell adhesion overnight, the cells were treated with serial dilutions of DESs dissolved in fresh HUVEC media. The plate was incubated for 4 hours before the media was aspirated and replaced with fresh media containing 20% MTS Reagent. After 1 hours the absorbance was measured at 490 nm (BioTek Synergy neo2).

To assess vascular permeability, transport across transwell cell culture experiments were used. First, sufficient 24 well‐plate transwell inserts were coated with 0.1% gelatin under sterile conditions and stored at 4 °C overnight. The excess gelatin was removed by inversion of transwell and washed with sterile PBS. HUVECs were cultured according to supplier protocols, subcultured, and seeded on the transwell inserts with 400 µL of 250 000 cells mL^−1^ media. The outer well was then filled with 600 µL of fresh media and allowed to grow for 48 hours before the experimental progression. After monolayers had formed, the media was removed from the top chamber and replaced with media containing 0.15% IL and 0.9 U mL^−1^ insulin, this was in preparation for the concentration and insulin to IL ratio to be used in the future in vivo studies. The plate was incubated in appropriate cell culture conditions and 300 µL samples were taken from the plate wells and replaced with fresh media every 10 minutes for 1 hour. The samples were then stored at 4 °C until they were diluted appropriately and quantified using ELISA.

### Formulation Stability in the Presence of Extracellular Matrix Proteins

To evaluate the physical stability of the formulations, the hydrodynamic size of insulin was measured using dynamic light scattering (DLS, Malvern Zetasizer Pro) in the presence of human collagen type I/III (Advanced BioMatrix, Carlsbad, CA). Insulin formulations with DESs, along with their respective clinical controls Humalog and saline, were added to a neutral‐pH solution of collagen at predetermined w/w ratios of collagen to insulin and size was measured at various timepoints.

Physical stability of the formulations in the presence of collagen was further assessed using fluorescence polarization (FP). In principle, a molecule of interest, in this case insulin around 5.8 kDa, will rotate faster in an unbound state than a bound state, in this case bound to collagen around 300 kDa, due to its Brownian rotation and its smaller molecular radius. When insulin is fluorescently labeled and excited with polarized light the perpendicular and parallel polarized light emission can be measured. Plate readers designed for FP can measure perpendicular and parallel polarized light to generate a polarization, with units mP, value which indicates the binding state of the fluorescently labeled protein. Bound insulin that is rotating more slowly will emit in the parallel direction and have a higher polarization value as the complex has not had sufficient time to rotate. Unbound insulin, or binding inhibition, is indicated by more perpendicular binding and lower polarization values.^[^ ^43^ ^]^ Formulations were prepared with Cy 5.5‐labeled insulin. The formulations were mixed with a collagen solution at predetermined w/w ratio, and fluorescence polarization was detected using Molecular Devices Flexstation 3 plate reader.

### Formulation Stability in the Presence of Human Serum

To assess insulin stability in the presence of human serum, formulations of the saline control and SPADE‐insulin were incubated at physiologically relevant concentrations (60 mU L^−1^)^[^ ^50^ ^]^ in human serum at 37 °C for 2 h. The insulin concentrations of the human serum, the saline control in human serum, and SPADE‐insulin in human serum were then measured using ELISA. The saline control and SPADE‐insulin concentrations were calculated by subtracting the human serum (without insulin spiked in) baseline. Theoretical concentrations were 60 mU L^−1^ which is indicative of 100% stability in human serum.

### In Vivo Pharmacokinetic and Bioavailability Experiments

All animal experiments were performed according to protocols approved by the Institutional Animal Care and Use Committee (IACUC) of Harvard University's Faculty of Arts and Sciences (FAS). The studies were performed in adult male Wistar non‐fasting rats weighing between 350 and 550 g (Charles River). The rats were anesthetized and blood was collected for *t* = 0 timepoint into K2 EDTA collection tubes. After initial blood collection, the rats received subcutaneous injections in the neck scruff. For the insulin study, the rats were dosed with 1 U kg^−1^ of Humalog or SPADE‐insulin formulation (both at a concentration of 3 U mL^−1^). Blood was then collected at 5, 10, 15, 20, 30, 45, 60, 150, and 240 minutes after injection. Insulin concentrations were then quantified with the relevant ELISA kits. The AUCs between each consecutive timepoint were calculated for each replicate using the trapezoid rule, the cumulative AUC at each timepoint was calculated by summing the AUCs that preceded the given time.

For the antibody study, rats received right flank subcutaneous injections of 10 mg kg^−1^. Blood samples on the first day were collected at 1, 3, 5, 8, and 11 hours after injection. The study continued and blood samples were collected once daily on days 1, 2, 3, 4, 7, 10, 14, 21, 28, 35, 42, and 49 after injection. Rituximab biosimilar concentrations were then quantified with ELISA. The AUCs between each consecutive timepoint was calculated for each replicate using the trapezoid rule, the cumulative AUC at each timepoint was calculated by summing the consecutive AUCs that preceded the given time.

### In Vivo Toxicity

Healthy female BALB/c mice (aged 7–8 weeks, Charles River) received a single subcutaneous injection of either DES solution or blank saline in the back scruff. Mice were euthanized at 1 and 7 days postinjection, and the local skin tissue (from stratum corneum to muscle) was harvested, fixed in paraformaldehyde, and sectioned for H&E staining. Another cohort of mice received four daily subcutaneous injections of either DES solution or blank saline, euthanized 24 hours post the last treatment, and blood and major organs were harvested. Blood was collected into both K2 EDTA coated tubes, for whole blood analysis, and clot activator coated tubes, for serum analysis. The clot activator tubes were centrifuged at 2600 rcf for 10 minutes to separate the serum from the clotted blood. Samples were kept on ice until hematology assays were run. After blood collection, mice were euthanized and vital organs were collected, washed with PBS, and fixed with 10% formalin. Whole blood and serum were analyzed for comprehensive complete blood count and blood chemistry (IDEXX BioAnalytics, North Grafton, MA), while organs were fixed and sectioned for H&E staining. All histological sections were imaged with AxioScan and interpreted by a professional histopathologist at the Rodent Histopathology Core at Harvard Medical School.

### Viscosity Measurements

Viscosity was measured similarly to previous methods.^[^ ^35^ ^]^ A steady‐state flow method using a TA Instruments HR 20 Discovery Hybrid Rheometer with a 40 mm diameter aluminum 2° cone was employed for measuring viscosity. 0.583 mL of formulation was applied to the bottom plate and temperature was equilibrated for 2 minutes at 25 °C. Shear rates from 1 to 1000 s^−1^ were swept through with 5 points per decade. This experiment was repeated 3 times for each of the formulations used using a fresh sample each time.

### Statistical Analysis

Unless otherwise specified, the data were plotted using GraphPad Prism 8 as mean ± standard error of the mean (SEM). Statistical significance was determined using unpaired parametric two‐tailed *t*‐tests in which the mean of each group was compared. Statistical significance for experimental results that had exactly two cohorts was assessed using a two‐tailed *t*‐test. Significance marks were categorized with the following *p*‐values: *p < 0.05, **p < 0.01, ***p < 0.001, ****p < 0.0001.

## Conflict of Interest

A.M.C., J.K., and S.M. are inventors on a patent application based on the results described in this manuscript (owned and managed by Harvard University). S.M. is an advisor/partner/board member/shareholder of Liquideon LLC, Cage Bio, i2o Therapeutics and inTumo Therapeutics.

## Supporting information

Supporting Information

## Data Availability

The data that support the findings of this study are available in the supplementary material of this article.
