# Supplementary material for: Deep Eutectic Solvents for Subcutaneous Delivery of Protein Therapeutics
Source: Adv Sci (Weinh). 2023 Jan 15;10(7):2205389. doi: 10.1002/advs.202205389 (PMC9982585; doi:10.1002/advs.202205389)
Supplement: Supplementary file 1 — Supporting Information [file ADVS-10-2205389-s001.pdf]

Supporting Information

Deep eutectic solvents for subcutaneous delivery of protein therapeutics

*Alexander M. Curreri, Jayoung Kim, Michael Dunne, Pavimol Angsantikul, Morgan Goetz, Yongsheng Gao, Samir Mitragotri**

Supplementary Text

NMR Results

Below are the 1D proton nuclear magnetic resonance results used to confirm the structures of the 10 deep eutectic solvents used in this study.

(2-hydroxyethyl)-N,N,N-trimethylammonium (choline) glycolate

^{1}H NMR (400 MHz, D2O) 3.26 (s, 9H, NC$\underline{H}_3$); 3.55 (m, 2H, NC$\underline{H}_2$CH$_2$OH); 4.09 (m, 2H, NCH$_2$C$\underline{H}_2$OH); 4.13 (m, 4H, HOC$\underline{H}_2$COO)

(2-hydroxyethyl)-N,N,N-trimethylammonium (choline) lactate

^{1}H NMR (400 MHz, D2O) 1.41 (m, 6H, C$\underline{H}_3$CHCOO); 3.20 (s, 9H, NC$\underline{H}_3$); 3.51 (m, 2H, NC$\underline{H}_2$CH$_2$OH); 4.05 (m, 2H, NCH$_2$C$\underline{H}_2$OH), 4.25 (qd, 2H, CH$_3$C$\underline{H}$COO)

(2-hydroxyethyl)-N,N,N-trimethylammonium (choline) propionate

^{1}H NMR (400 MHz, D2O) 1.07 (t, 6H, C$\underline{H}_3$CH$_2$COO); 2.28 (q, 4H, CH$_3$C$\underline{H}_2$COO); 3.19 (s, 9H, NC$\underline{H}_3$); 3.51 (m, 2H, NC$\underline{H}_2$CH$_2$OH); 4.06 (m, 2H, NCH$_2$C$\underline{H}_2$OH)

(2-hydroxyethyl)-N,N,N-trimethylammonium (choline) hexenoate

^{1}H NMR (400 MHz, DMSO)
0.91 (t, 6H, OOCCHCHCH$_2$CH$_2$C$\underline{H}_3$); 1.47 (m, 4H, OOCCHCHCH$_2$C$\underline{H}_2$CH$_3$);
2.19 (qd, 4H, OOCCHCHC$\underline{H}_2$CH$_2$CH$_3$); 3.20 (s, 9H, NC$\underline{H}_3$); 3.51 (m, 2H, NC$\underline{H}_2$CH$_2$OH); 4.06 (m, 2H, NCH$_2$C$\underline{H}_2$OH); 5.85 (dt, 2H, OOCCHC$\underline{H}$CH$_2$CH$_2$CH$_3$); 6.86 (dt, 2H, OOCC$\underline{H}$CHCH$_2$CH$_2$CH$_3$)

(2-hydroxyethyl)-N,N,N-trimethylammonium (choline) geranate

^{1}H NMR (400 MHz, DMSO)

1.57 (s, 6H, $OOCCHC(CH_3)CH_2CH_2CHC(CH_3)C\underline{H}_3$); 1.64 (s, 6H, $OOCCHC(CH_3)CH_2CH_2CHC(C\underline{H}_3)CH_3$); 2.05 (m, 14H, $OOCCHC(C\underline{H}_3)C\underline{H}_2C\underline{H}_2CHC(CH_3)CH_3$); 3.11 (s, 9H, $NC\underline{H}_3$); 3.42 (m, 2H, $NC\underline{H}_2CH_2OH$); 3.85 (m, 2H, $NCH_2C\underline{H}_2OH$); 5.07 (t, 2H, $OOCCHC(CH_3)CH_2CH_2C\underline{H}C(CH_3)CH_3$); 5.57 (s, 2H, $OOCC\underline{H}C(CH_3)CH_2CH_2CHC(CH_3)CH_3$)

(2-acetoxy)-N,N,N-trimethylammonium (acetylcholine) glycolate

^{1}H NMR (400 MHz, D2O) 2.11 (s, 3H, $NCH_2CH_2OCOC\underline{H}_3$); 3.53 (s, 9H, $NC\underline{H}_3$); 4.08 (m, 4H, $HOC\underline{H}_2COO$); 4.22 (m, 2H, $NC\underline{H}_2CH_2OCOCH_3$); 4.58 (m, 2H, $NCH_2C\underline{H}_2OCOCH_3$)

(2-acetoxy)-N,N,N-trimethylammonium (acetylcholine) lactate

^{1}H NMR (400 MHz, D2O) 1.43 (m, 6H, $C\underline{H}_3CHCOO$); 2.12 (s, 3H, $NCH_2CH_2OCOC\underline{H}_3$); 3.50 (s, 9H, $NC\underline{H}_3$); 4.20-4.30 (m, 4H, $NC\underline{H}_2CH_2OCOCH_3$ & $CH_3C\underline{H}COO$); 4.61 (m, 2H, $NCH_2C\underline{H}_2OCOCH_3$)

(2-acetoxy)-N,N,N-trimethylammonium (acetylcholine) propionate

^{1}H NMR (400 MHz, D2O) 1.12 (m, 6H, $C\underline{H}_3CH_2COO$); 2.18 (s, 3H, $NCH_2CH_2OCOC\underline{H}_3$); 2.28 (m, 4H, $CH_3C\underline{H}_2COO$); 3.58 (s, 9H, $NC\underline{H}_3$); 4.25 (m, 2H, $NC\underline{H}_2CH_2OCOCH_3$); 4.64 (m, 2H, $NCH_2C\underline{H}_2OCOCH_3$)

(2-acetoxy)-N,N,N-trimethylammonium (acetylcholine) hexenoate

^{1}H NMR (400 MHz, DMSO)
0.89 (m, 6H, $OOCCHCHCH_2CH_2C\underline{H}_3$); 1.50 (m, 4H, $OOCCHCHCH_2C\underline{H}_2CH_3$); 2.09-2.23 (m, 7H, $NCH_2CH_2OCOC\underline{H}_3$ & $OOCCHCHC\underline{H}_2CH_2CH_3$); 3.55 (s, 9H, $NC\underline{H}_3$); 4.18 (m, 2H, $NC\underline{H}_2CH_2OCOCH_3$); 4.60 (m, 2H, $NCH_2C\underline{H}_2OCOCH_3$); 5.82 (m, 2H, $OOCCHC\underline{H}CH_2CH_2CH_3$); 6.83 (m, 2H, $OOCC\underline{H}CHCH_2CH_2CH_3$)

(2-acetoxy)-N,N,N-trimethylammonium (acetylcholine) geranate

^{1}H NMR (400 MHz, DMSO)
1.58-1.65 (d, 12H, $OOCCHC(CH_3)CH_2CH_2CHC(C\underline{H}_3)C\underline{H}_3$); 2.05-2.13 (m, 17H, $OOCCHC(C\underline{H}_3)C\underline{H}_2C\underline{H}_2CHC(CH_3)CH_3$ & $NCH_2CH_2OCOC\underline{H}_3$); 3.54 (s, 9H, $NC\underline{H}_3$); 4.16 (m, 2H, $NC\underline{H}_2CH_2OCOCH_3$); 4.53 (m, 2H, $NCH_2C\underline{H}_2OCOCH_3$); 5.10 (t, 2H, $OOCCHC(CH_3)CH_2CH_2C\underline{H}C(CH_3)CH_3$); 5.63 (s, 2H, $OOCC\underline{H}C(CH_3)CH_2CH_2CHC(CH_3)CH_3$)

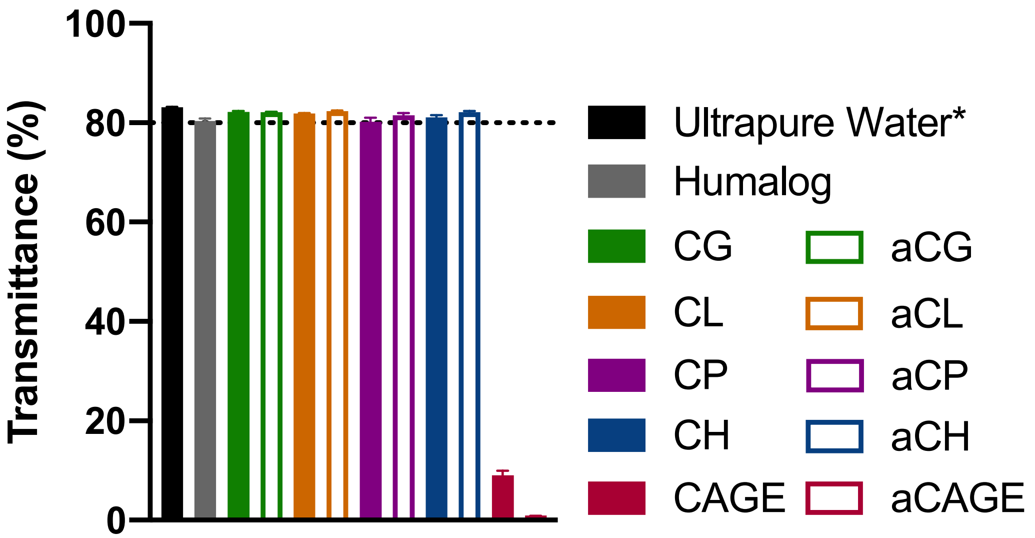

Figure S1.

Transmittance (%) for insulin-DES formulations and relevant controls. The dotted line marks that 80% transmittance that was used as the minimum threshold to proceed to next study.

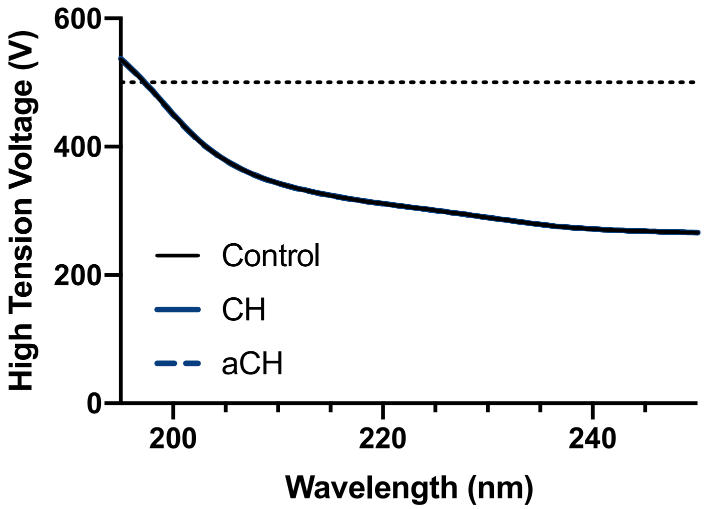

Figure S2.

High tension voltage (V) vs. wavelength that was measured for control (fresh stable insulin stock), CH, and aCH formulations during CD experiments. The 500 V maximum threshold (marked by the dotted line) is used as a check to confirm CD spectra integrity.

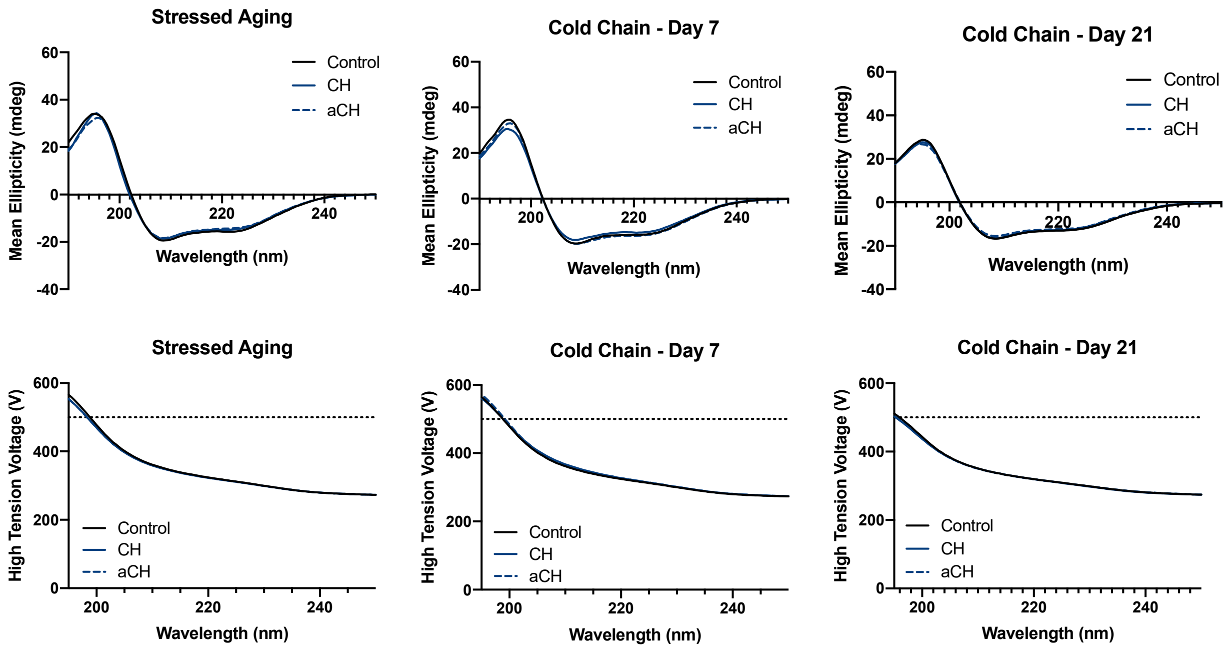

Figure S3.

Circular dichroism for stressed aging (rotational shaking at 37°C for 48 hours) and cold chain (4°C for 7 and 21 days) of control (fresh, stable insulin stock), CH, and aCH formulations. The corresponding high tension voltage (V) vs. wavelength are shown below the relevant plots. The 500 V maximum threshold (marked by the dotted line) is used as a check to confirm CD spectra integrity.

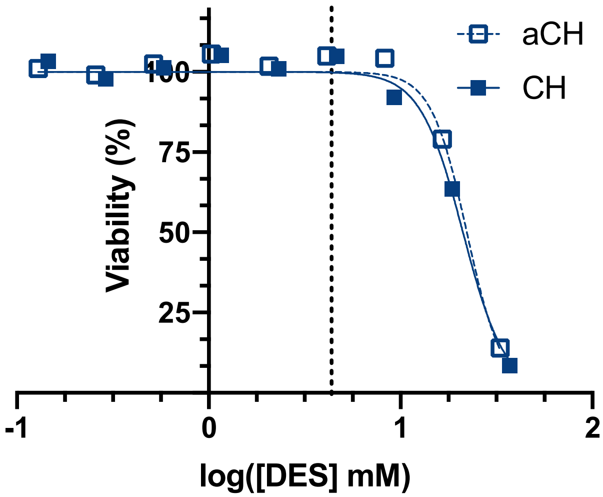

Figure S4.

Cell viability (%) vs logarithm of DES concentration (mM). The dotted line represents the maximum viable concentration (0.15%).

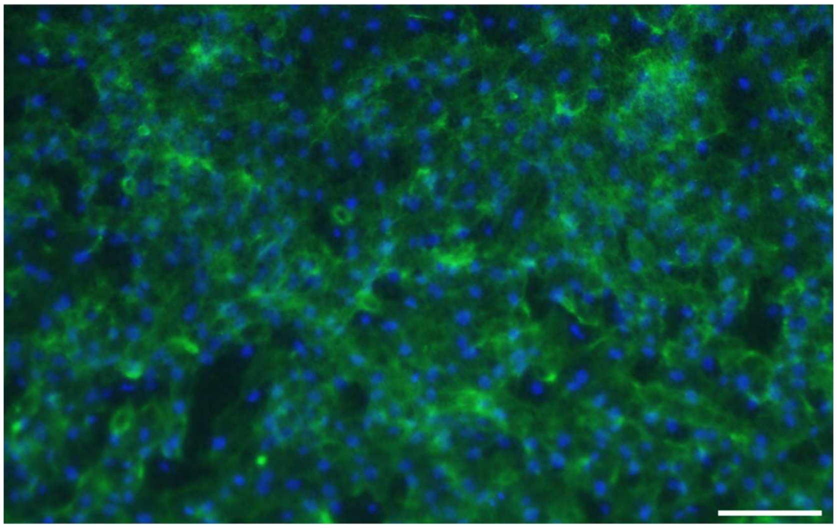

Figure S5.

Fluorescent images of transwell HUVEC monolayer, cells were fixed and the nuclei were stained with Hoechst 33342 (blue) and cytoplasm was stained with ActinGreen 488 (green). Scale bar, 100 μm.

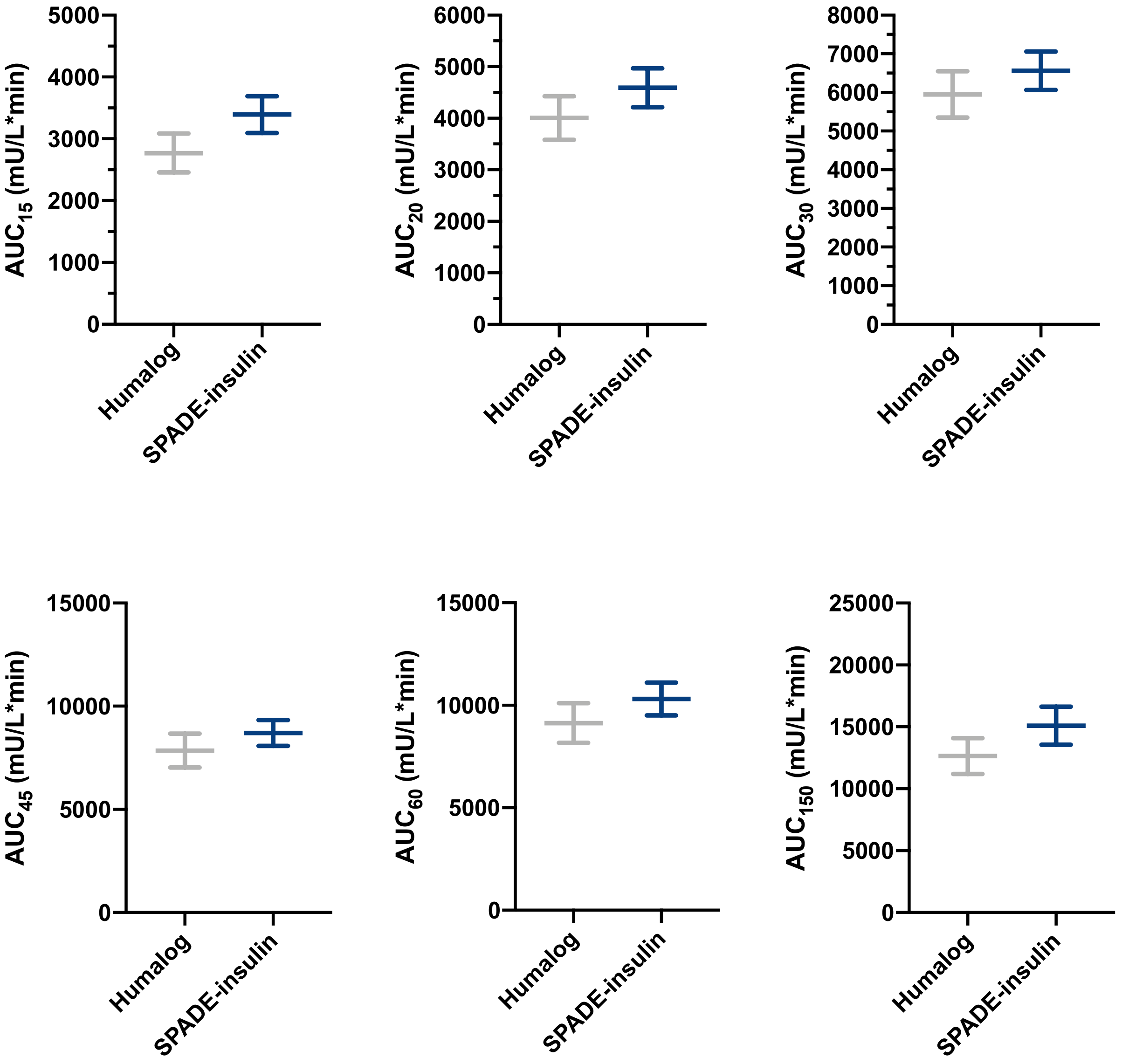

Figure S6.

Area under the curve (AUC) values for the insulin pharmacokinetic study at timepoints between 15 and 150 minutes.

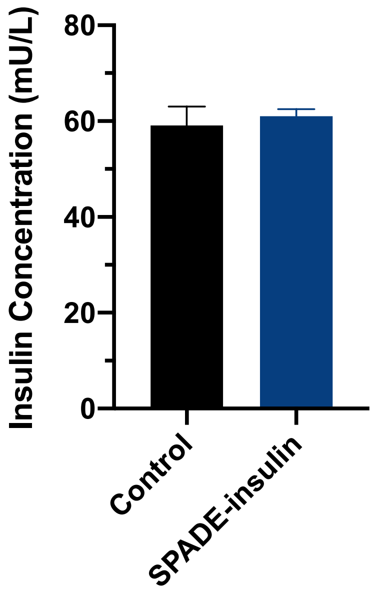

Figure S7.

SPADE-insulin (n=3) concentration measured using ELISA in human serum compared with the control (insulin formulated in saline, n=3).

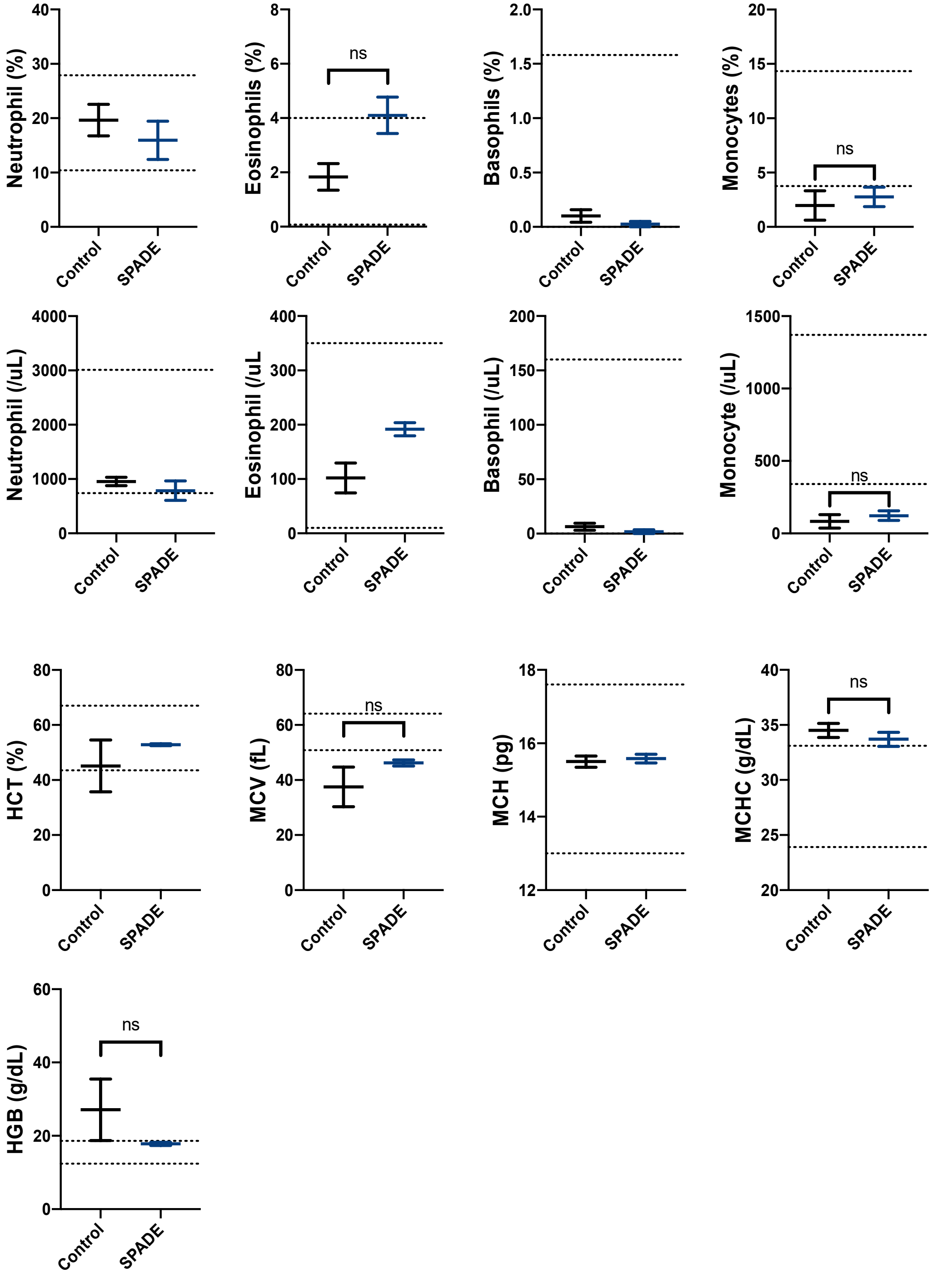

Figure S8.
Additional whole blood analysis results comparing control (saline) and SPADE, including various leukocyte levels, hematocrit (HCT), mean corpuscular volume (MCV), mean corpuscular hemoglobin (MCH), mean corpuscular hemoglobin concentration (MCHC), and hemoglobin (HGB). Statistical significance was determined with t-test. *$p < 0.05$, **$p < 0.01$, ***$p < 0.001$, ****$p < 0.0001$.

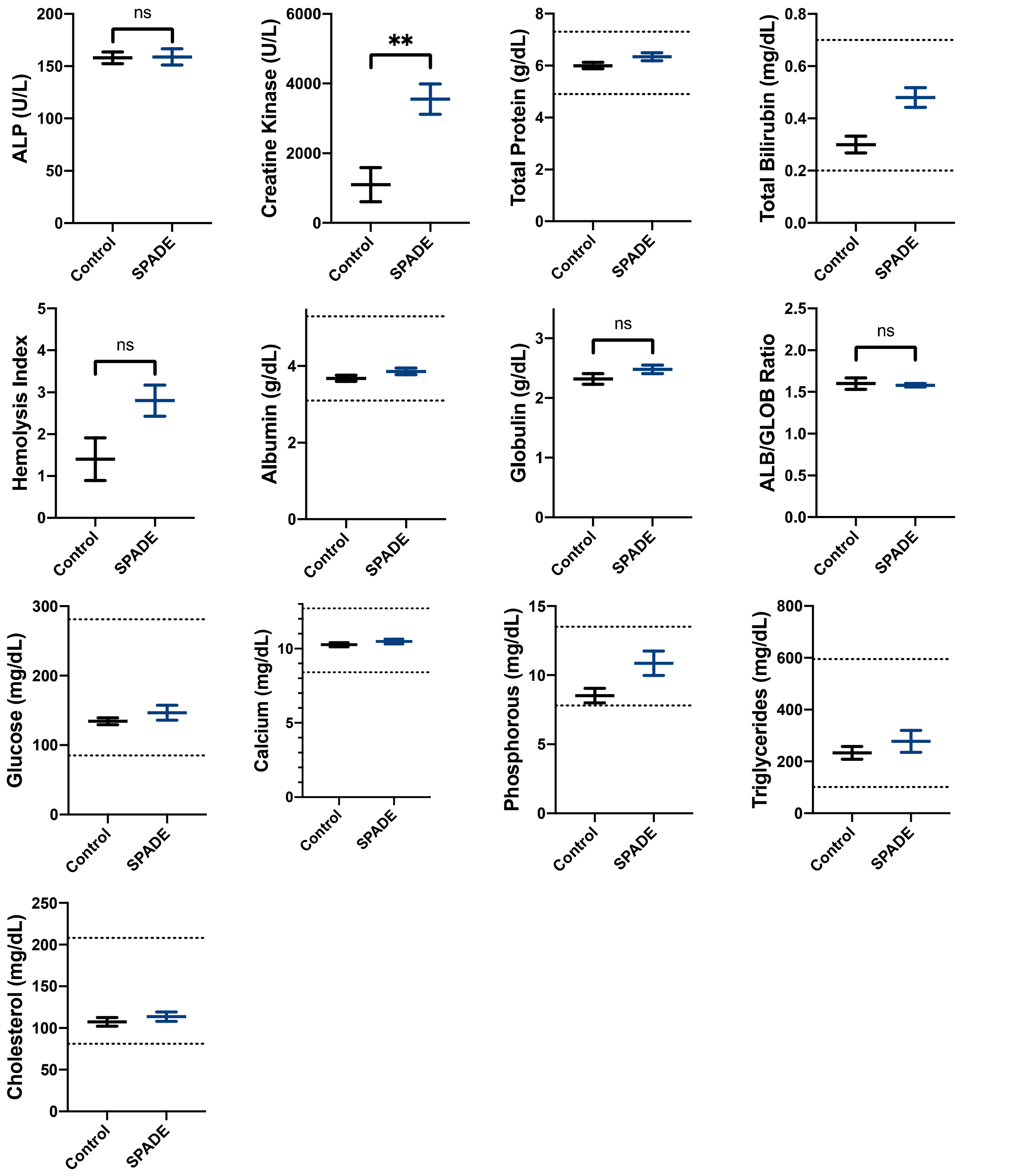

Figure S9.
Additional serum analysis results comparing control (saline) and SPADE, including a variety of serum proteins, enzymes, alkaline phosphatase (ALP), ions, and other biomarkers. Only creatine kinase levels were significantly higher in the SPADE group, however this is likely due the release associated with cardiac puncture that was performed on some mice in the group. Statistical significance was determined with t-test. *$p < 0.05$, **$p < 0.01$, ***$p < 0.001$, ****$p < 0.0001$.

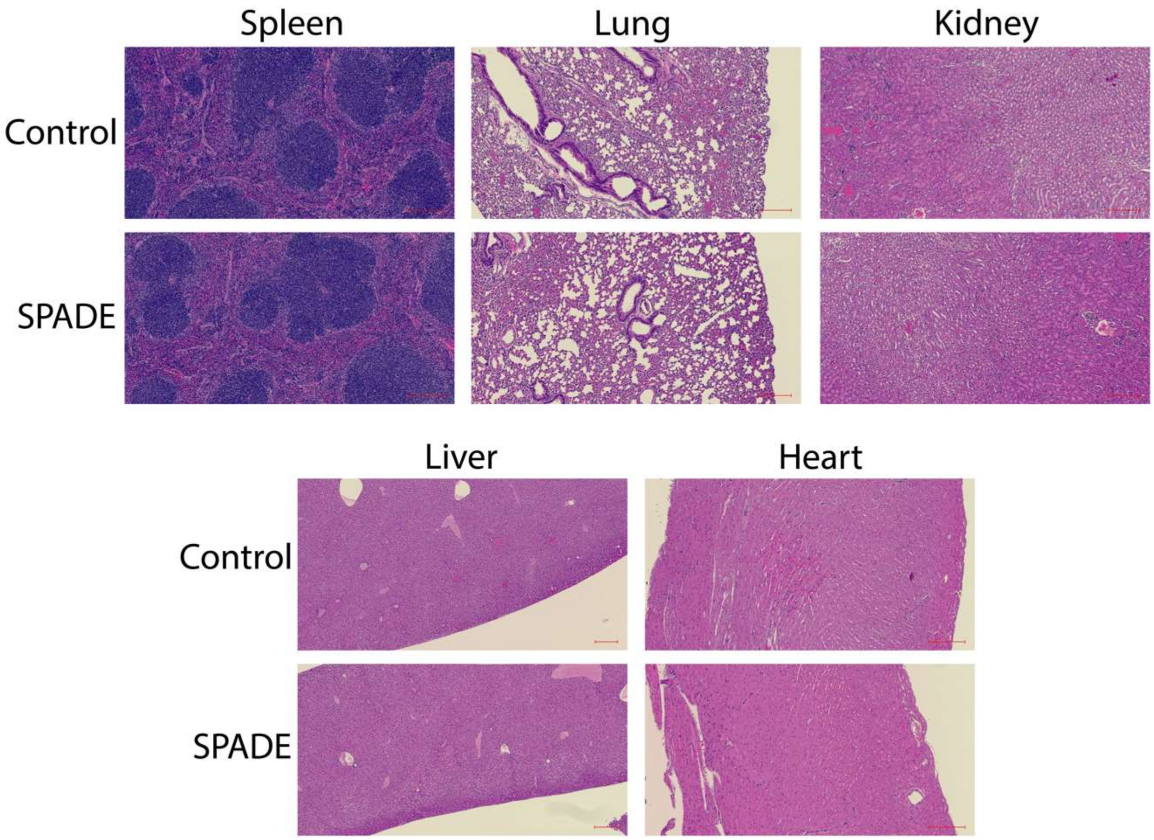

Figure S10.

H&E staining of vital organs from the toxicity study in which mice were dosed multiple times with control (saline) and SPADE. Scale bars, 200 μm.

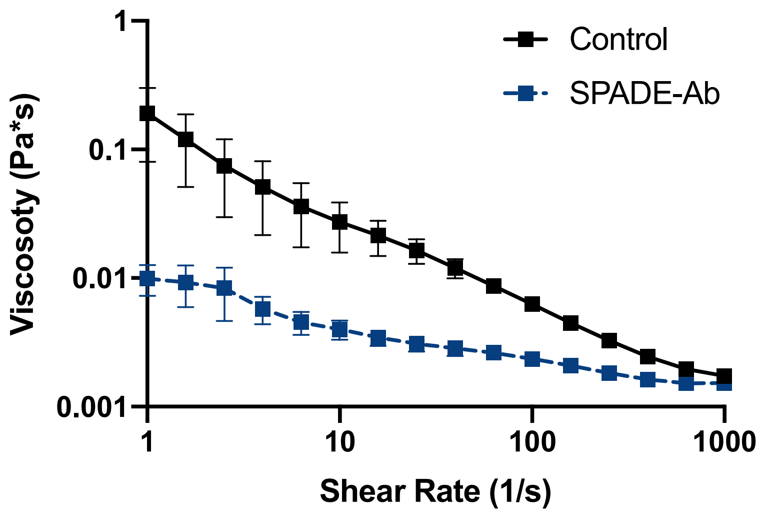

Figure S11.

Viscosity measurements of control (antibody formulated in saline) and SPADE-Ab formulations over shear rate rage of 1 to 1000 s^{-1}).

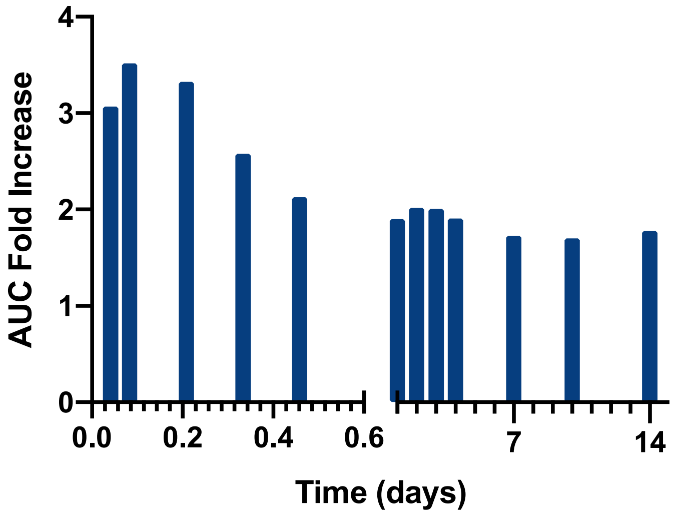

Figure S12.

Fold-change of AUC (AUC-SPADE-mAb/AUC-Control) for the first 14 days of the rituximab study.

Acid Name	pKa	log(P)	MW (g/mol)	Carbon Chain
**Glycolic**	3.83	-1.11	76.06	2
**Lactic**	3.86	-0.72	90.08	3
**Propionic**	4.33	0.33	74.08	3
**Hexenoic**	4.74	1.81	114.14	6
**Geranic**	5.26	2.82	168.23	8

Table S1.
Table of DES anion properties.

	Time	Serum Concentration	AUC
Hours	1	<0.0001****	<0.0001****
	2	0.0029***	0.0003***
	5	0.0074**	0.0025**
	8	0.0261*	0.0062**
	11	0.0512	0.0109*
Days	1	0.0263*	0.0205*
	2	0.022*	0.02*
	3	0.0164*	0.0152*
	4	0.0498*	0.0162*
	7	0.0826*	0.0288*
	10	0.0193*	0.0279*
	14	0.0288*	0.0215*
	21	N/A	0.0159*
	28	N/A	0.027*
	35	N/A	0.0335*
	42	N/A	0.0368*
	49	N/A	0.0397*

Table S2.

Table of statistical significance values (p-value) of the t-tests performed on the serum concentrations and area under the curve (AUC) values for all timepoints in the SPADE-mAb bioavailability study. Significance marks were categorized with the following p-values: *$p < 0.05$, **$p < 0.01$, ***$p < 0.001$, ****$p < 0.0001$.
